# A chemical screen identifies two novel small compounds that alter *Arabidopsis thaliana* pollen tube growth

**DOI:** 10.1186/s12870-019-1743-9

**Published:** 2019-04-22

**Authors:** Ferdousse Laggoun, Flavien Dardelle, Jérémy Dehors, Denis Falconet, Azeddine Driouich, Christophe Rochais, Patrick Dallemagne, Arnaud Lehner, Jean-Claude Mollet

**Affiliations:** 1 Normandie Université, UNIROUEN, Laboratoire Glycobiologie et Matrice Extracellulaire Végétale EA4358, Fédération de Recherche “NORVEGE”- FED 4277, 76000 Rouen, France; 2 Laboratoire de Physiologie Cellulaire et Végétale, CNRS, CEA, INRA, Université Grenoble Alpes, Institut de Biosciences et Biotechnologies de Grenoble, CEA Grenoble, 38000 Grenoble, cedex 9 France; 3 Normandie Université, UNICAEN, Centre d’Etudes et de Recherche sur le Médicament de Normandie, CNRS 3038 INC3M, SFR ICORE, 14032, Caen, France; 4 Present Address: LPS-BioSciences, Bâtiment 409, Université Paris-Sud, 91400 Orsay, France

**Keywords:** Pollen tubes, Chemical screen, Callose, Cell wall deposition, Pectins, ROS, Tip-polarized growth, RIC4, Actin dynamics

## Abstract

**Background:**

During sexual reproduction, pollen grains land on the stigma, rehydrate and produce pollen tubes that grow through the female transmitting-tract tissue allowing the delivery of the two sperm cells to the ovule and the production of healthy seeds. Because pollen tubes are single cells that expand by tip-polarized growth, they represent a good model to study the growth dynamics, cell wall deposition and intracellular machineries. Aiming to understand this complex machinery, we used a low throughput chemical screen approach in order to isolate new tip-growth disruptors. The effect of a chemical inhibitor of monogalactosyldiacylglycerol synthases, galvestine-1, was also investigated. The present work further characterizes their effects on the tip-growth and intracellular dynamics of pollen tubes.

**Results:**

Two small compounds among 258 were isolated based on their abilities to perturb pollen tube growth. They were found to disrupt in vitro pollen tube growth of tobacco, tomato and *Arabidopsis thaliana.* We show that these 3 compounds induced abnormal phenotypes (bulging and/or enlarged pollen tubes) and reduced pollen tube length in a dose dependent manner. Pollen germination was significantly reduced after treatment with the two compounds isolated from the screen. They also affected cell wall material deposition in pollen tubes. The compounds decreased anion superoxide accumulation, disorganized actin filaments and RIC4 dynamics suggesting that they may affect vesicular trafficking at the pollen tube tip.

**Conclusion:**

These molecules may alter directly or indirectly ROP1 activity, a key regulator of pollen tube growth and vesicular trafficking and therefore represent good tools to further study cellular dynamics during polarized-cell growth.

**Electronic supplementary material:**

The online version of this article (10.1186/s12870-019-1743-9) contains supplementary material, which is available to authorized users.

## Background

During sexual reproduction, pollen grains land on the stigma, rehydrate and produce pollen tubes that grow through the female transmitting-tract tissue allowing a proper delivery of the two sperm cells to the ovule [1]. During this journey, the pollen tube perceives different signals promoting its growth, adhesion and guidance [2–5].

Pollen tube is one of the fastest tip-growing cells. They can reach growth rate from 58 to 400 nm.sec^− 1^, depending on the species [6]. This implies an extremely efficient system for delivering and modifying membrane and cell wall material at the tip, which is highly coordinated for pollen tube oscillatory growth [7, 8]. Oscillatory growth is followed by an intracellular tip-high gradient of calcium. In addition, calcium is an important second messenger that plays a key role in the regulation of pollen tube elongation and guidance [9–11]. ROS (reactive oxygen species) are also involved in pollen tube initiation, polarized-growth and elongation. Despite their toxicities, ROS act as a second messenger and are localized at the pollen tube tip [12–14]. ROS production is partially associated with RBOH (respiratory burst oxidase homologs) a family of NADP(H) oxidases localized at the plasma membrane [12, 15].

During pollen tube growth, the cytoskeleton, mainly composed of actin microfilaments and microtubules, maintains the cytoplasm movements inside the pollen tube as the so-called “reverse fountain streaming”. Microtubules are involved in the male germ unit (MGU) movement whereas actin microfilaments are implicated in organelle and vesicular trafficking as well as pollen tube growth [6]. The use of live-cell actin markers such as Lifeact-EGFP has confirmed the involvement and the spatial distribution of actin cables during pollen tube growth [16, 17]. In the shank of pollen tubes, actin filaments follow an axial arrangement which allows organelle and vesicle transport to the tip. At the tip, actin filaments form a regular structure usually called actin fringe [16, 18–21]. The actin fringe may contribute to the pectin-focused secretion in the apical cell wall by directing vesicles to specific sites of fusion at the extreme tube apex and contributing to the polarized growth of pollen tubes [22].

At the center of this process, ROP (RhO-related in Plant), a unique sub-family of Rho-GTPases in plants, is known to regulate tip-growth and polar cell expansion in different cell types, particularly the pollen-specific ROP1, is an essential regulator for pollen tip-growth [23–26]. ROP proteins are cytosolic and inactive in the GDP-bound state and are active when associated with the plasma membrane in the GTP-bound state. Guanine nucleotide exchange factors (GEFs) catalyze GDP release exchanged with GTP and GTPase activating proteins (GAPs) enhance GTP hydrolysis, inducing ROP inactivation [27]. ROP1 is localized in the plasma membrane of the pollen tube tip and has an oscillatory interaction with two downstream targets that are CRIB motif-containing ROP-interacting proteins (RIC3 and RIC4) [16, 28]. These two proteins have two distinct functions and control two different pathways downstream of ROP1: the formation of a tip-focused calcium gradient and the assembly of the actin fringe, respectively [28, 29].

Vesicles containing cell wall materials are transported by the reverse fountain streaming [30]. In *Arabidopsis thaliana*, pollen tubes possess a specific cell wall organization compared to somatic cells. In the shank, it is composed of an inner cell wall layer enriched in β-glucan (mostly callose and minor amount of cellulose) and an outer layer composed of cellulose, hemicellulose (mostly xyloglucan) and pectins including weakly methylesterified homogalacturonan (HG) and rhamnogalacturonan-1 [31, 32]. Callose is not only present in the inner cell wall layer but also in plugs that are regularly synthesized allowing the vegetative cell to conserve a regular volume during pollen tube elongation [33, 34]. The mode and pattern of callose plug deposition vary among the species [35, 36]. At the tip, only one cell wall layer is present and is enriched in methylesterified HG rhamnogalacturonan-1, arabinogalactan proteins (AGPs) and xyloglucan while it contains little amounts of cellulose [31, 32].

Despite the central role of pollen tubes during sexual plant reproduction leading to the production of high numbers of healthy seeds, this tip-growing cell represents also a very good model for studying polarized growth and cell wall synthesis and remodeling [8, 32, 37–40].

For many years, different approaches were used to understand the complex processes involved in pollen tube growth and cell wall remodeling including functional genomics, enzyme and pharmacological treatments [41–44]. Recently, chemical screens of small compounds found significant applications in plant cell biology, mostly in relation with hormonal signaling (e.g., auxin, brassinosteroid or strigolactone) [45–48], and plant-pathogen interactions [49, 50]. However only few studies were conducted on pollen. An automated image-based screen was developed for compounds that inhibited pollen germination in vitro or affected polar growth [51]. Pollen tubes were also used to understand plant growth and development [52] or cellular processes such as endomembrane trafficking [53].

In the present study, we screened 258 diverse compounds from the chemical library from CERMN (Centre d’Etudes et de Recherche sur le Médicament de Normandie, Normandie Univ, UniCaen, France), part of la Chimiothèque Nationale (http://chimiotheque-nationale.cn.cnrs.fr/) on *A. thaliana* pollen germination and pollen tube growth. Two compounds t we named Disruptol-A and Disruptol-B were isolated. Together with galvestine-1, known to alter pollen tube growth and inhibit the biosynthesis of galactolipids through inhibition of monogalactosyldiacylglycerol (MGDG) synthases [54], these molecules were able to interfere with pollen germination and disrupt the polarized growth of the pollen tube in a dose-dependent manner by modulating actin dynamics and ROS accumulation. The distribution of cell wall polymers including callose, pectins and arabinogalactan-proteins (AGPs) was also affected by the treatments suggesting that the compounds may directly or indirectly perturb vesicular trafficking at the pollen tube tip. Their dose-dependent effects point out the potential benefits of these compounds as new tools to study polarized growth.

## Results

### Chemical screen identified two compounds from the CERMN chemical library

**Fig. 1:**
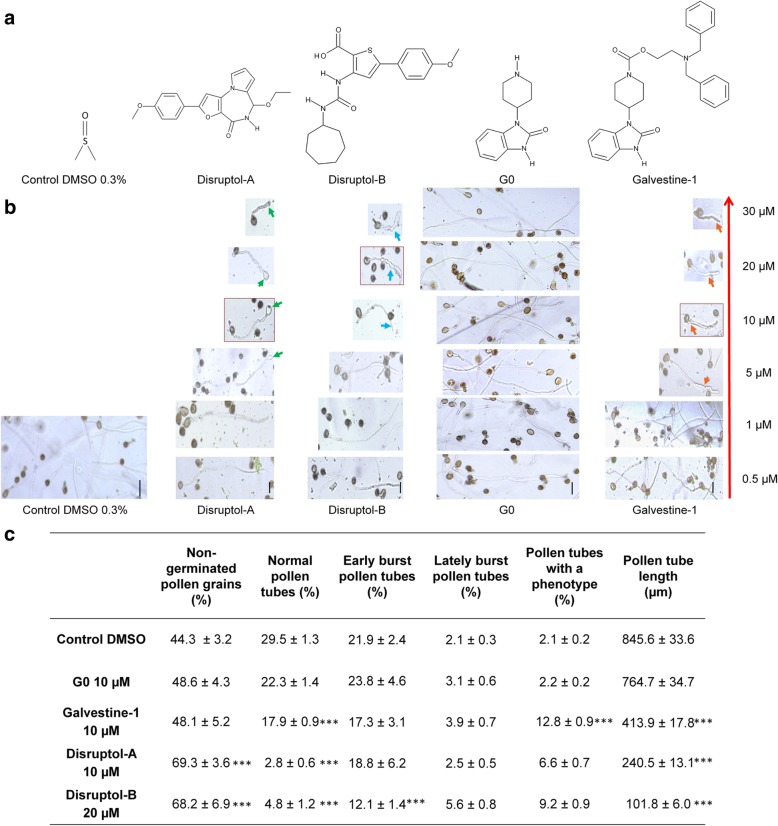
Dose-response effect of the compounds on *Arabidopsis thaliana* pollen tubes. **a** Structure of the compounds selected during the chemical screen and further investigated in the present study: Disruptol-A, Disruptol-B, Galvestine-1 and the negative control of galvestine-1: G0. **b** Dose-response effect of the compounds on pollen tube phenotype. **c** Effect of the selected concentration of the compounds after 6 h of culture on pollen germination, phenotype rates and pollen tube length. Data represent the mean of (665 ≤ *n* ≤ 1249) pollen for pollen germination and phenotype or (34 ≤ *n* ≤ 76 pollen tubes) for pollen tube length from two biological replicates ± SEM (Standard Error of the Mean). Asterisks indicate significant differences (*** *P* < 0.001, ** *P* < 0.01 and * *P* < 0.05) from the control DMSO 0.3% according to pairwise comparison using Dunnett’s test analysis with R software. Red framed Images indicate the selected concentrations. Green arrows indicate bulging pollen tubes. Blue arrows indicate misshaped pollen tubes. Orange arrows indicate shorter pollen tubes. Scale bar = 40 μm

Among the 258 diverse compounds tested at 20 μM during the primary screen, two compounds were selected based on their abilities to distrupt the tip-polarized growth of *A. thaliana* pollen tubes. The compounds were named Disruptol-A and Disruptol-B (Fig. 1a). Even if Disruptol-A is a tricyclic furopyrrolodiazepinone and Disruptol-B a linear ureidothiophenecarboxylic acid, the two derivatives share common structural features (anisole pending ring, five-membered heterocycle, carboxamide or ureido group, alkyl or cycloalkyl chain...) and Disruptol-A can be considered as a rigidified analog of Disruptol-B. Another compound (galvestine-1) was also used in this study (Fig. 1a). Galvestine-1 was shown to reduce pollen tube length in vitro [54] and was further characterized. The negative control of galvestine-1 is G0. G0 molecule possesses a galvestine-1 structure modification that leads to the loss of bioactivity (Fig. 1a) [54].

### Effects of the compounds on pollen germination and pollen tube growth

The effect of the selected compounds on pollen germination and pollen tube growth was investigated in 96-well plates using Arabidopsis, tobacco and tomato pollen grains incubated for 6 h with the compounds at different concentrations (Fig. 1b, Additional file 1: Figure S1). Pollen tubes treated with 0.3% DMSO (control) had an average length of 845.6 μm ± 33.6 (Fig. 1c). Similar results were obtained with different concentrations of the G0 (galvestine negative control). Both DMSO and G0 treatments did not show any changes neither on pollen germination nor on the shape and/or the length of the pollen tubes (Fig. 1b and c). The rates of normal pollen tubes ranged from 22.3% ± 1.4 to 29.5% ± 1.3 for G0 and the 0.3% DMSO, respectively and only 2% of pollen tubes displayed abnormal shape (Fig. 1c). Disruptol-A, Disruptol-B and galvestine-1 had a dose-dependent effect on pollen tube length (Fig. 1b and c). After 6 h, pollen tubes treated with Disruptol-A were shorter (Fig. 1c) and displayed a swollen tip above 5 μM (Fig. 1b). The tubes reached only 400 μm after 6 h, half the length of the DMSO control (Fig. 1c). In the presence of Disruptol-B, pollen tubes were also shorter above 5 μM (Fig. 1b). At 20 μM, pollen tubes were misshaped at the tip (Fig. 1b). Galvestine-1 also caused a significant reduction in pollen tube length at 10 μM (Fig. 1c) but did not induce any strong deformation (Fig. 1b). Similar effects were observed on treated *Solanum lycopersicum* and *Nicotiana tabacum* pollen tubes (Additional file 1: Figure S1). To investigate further the effects of the compounds, only one concentration was selected and used for each based on the ratio between germination rates and the frequency of the phenotypes. Thus, the concentrations were selected to avoid severe effects (non-germinated, non-growing or dead pollen tubes) but to have viable and growing tubes with a phenotype. The selected concentrations (highlighted by red squares in Fig. 1b) were 10 μM for Disruptol-A and galvestine-1 and 20 μM for Disruptol-B.

The early burst pollen tubes (i.e. pollen tubes bursting just after germination) did not change dramatically (22–24% in the controls and 17–19% with galvestine-1 and Disruptol-A except for Disruptol-B (12%) (Fig. 1c). Treatment with 10 μM galvestine-1 resulted in shortened pollen tube (12.8%) and anincrease of the proportion of lately burst pollen tubes (i.e. burst of pollen tubes with a length equal to the pollen grain diameter ~ 20 μm) (3.9%) compared to the control G0. Treatments with Disruptol-A (10 μM) and Disruptol-B (20 μM) affected pollen tube germination. The rates of non-germinated pollen grains were 69.3% ± 3.6 with Disruptol-A and 68.2% ± 6.9 with Disruptol-B significantly higher than those of the control samples (44.3% ± 3.2). Similarly, the rates of normal pollen tubes with Disruptol-A and Disruptol-B were 2.8% ± 0.6 and 4.8% ± 1.2, respectively and significantly lower than in the control (29.5% ± 1.3) (Fig. 1c).

**Fig. 2:**
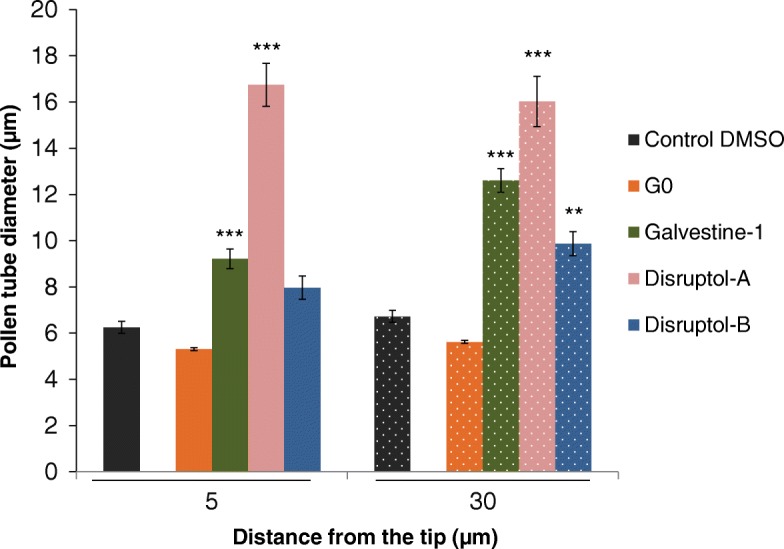
Effect of the selected concentrations of the compounds on pollen tube diameters at 5 μm and 30 μm from the tip. Data represent the mean of 15 pollen tubes from two biological replicates ± SEM. Asterisks indicate significant differences (*** *P* < 0.001, ** *P* < 0.01 and * *P* < 0.05) from the control DMSO 0.3% according to Dunnett’s test analysis with R software

Finally, all the compounds have a significant effect on the pollen tube diameter. The diameters of the tubes were measured at 5 and 30 μm back from the tip (Fig. 2). The pollen tube diameters in the controls were between 5 and 7 μm (Fig. 2). After 6 h of incubation with galvestine-1, pollen tube diameters increased significantly at 5 μm and 30 μm from the tip, reaching 9.2 μm ± 0.4 and 12.6 μm ± 0.5, respectively. Disruptol-B treatment induced also a significant increase of the pollen tube diameters but only at 30 μm from the tip (9.9 μm ± 0.5). Disruptol-A has the most important effect on the diameters of pollen tubes reaching around 16 μm at 5 and 30 μm back from the tip (Fig. 2), due to the swelling of the tip (Fig. 1b).

### Effect of the compounds on superoxide production

**Fig. 3:**
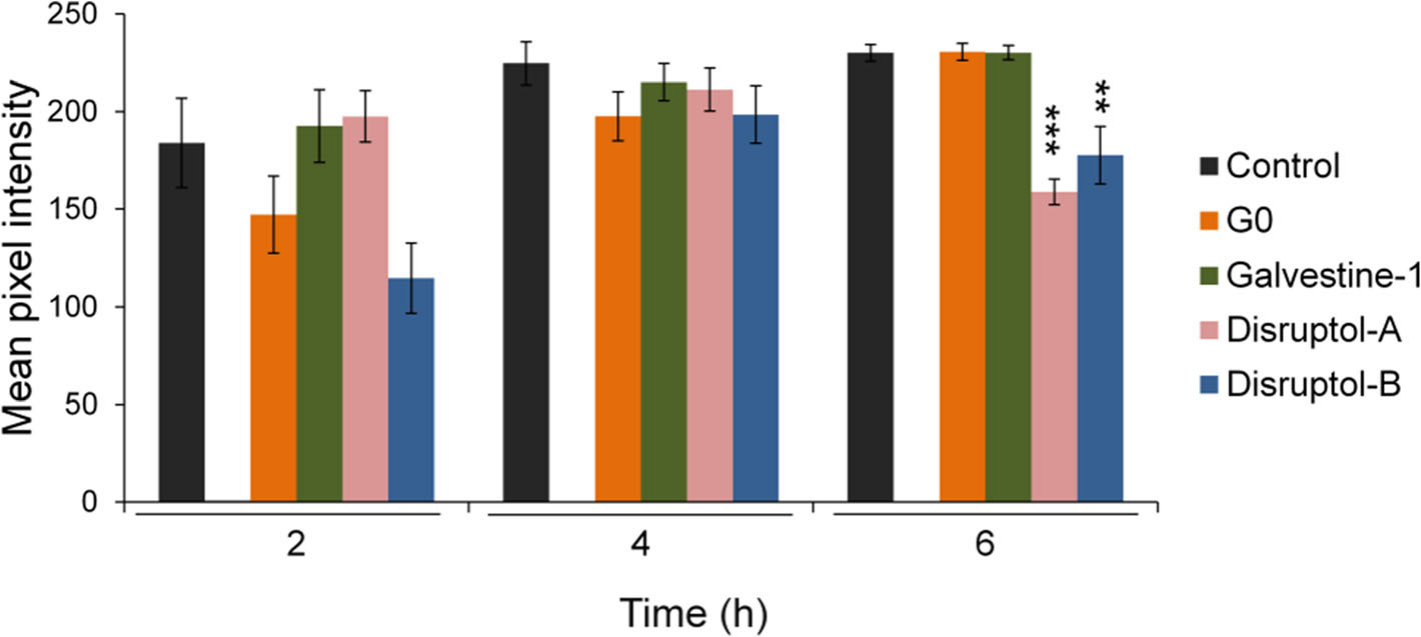
Effect of the compounds on anion superoxide (O2^•-^) production. Mean pixel intensity of NBT staining after 2, 4 and 6 h of treatment. For mean pixel intensity, pictures were transformed as grayscale images, a mark was drawn from the tip to 30 μm behind and the region of Interest, ROI, was manually selected using freehand selection tool. The mean value of the pixel intensity was obtained with the measure tool of the Image J software. Data represent the mean of (6 ≤ *n* ≤ 14 pollen tubes) from 2 biological replicates ± SEM. Asterisks indicate significant differences (*** *P* < 0.001, ** *P* < 0.01 and * *P* < 0.05) from the control DMSO 0.3% according to pairwise comparison using Dunnett’s test analysis (for 2 h and 4 h) and according to pairwise comparison using Wilcoxon’s test analysis with Holm adjustment (for 6 h) with R software according to parametric and non-parametric batch of time dataset

To determine the effect of the compounds on superoxide production, we used NBT staining. NBT allows to detect the superoxide anion (O_2_^•-^) after 20 min of incubation with the reagent. NBT staining was quantified using Image J software as the mean pixel intensity in the pollen tube tip (Fig. 3). Under the two control conditions (DMSO and G0), the accumulation of superoxide regularly increased after 2, 4 and 6 h (Fig. 3) and the pixel mean intensity slightly increased over time: 184, 224 and 230 after 2, 4 and 6 h respectively for the DMSO and 150, 197 and 230 after 2, 4 and 6 h respectively for G0 (Fig. 3). Galvestine-1 induced the same increase with no significant difference compared to the control (Fig. 3). Treatments with Disruptol-A and Disruptol-B showed no significant differences after 2 and 4 h when compared to the DMSO (Fig. 3). However, after 6 h, the anion superoxide production was significantly lower in the treated samples (158.7 ± 0.12 for Disruptol-A, 177.5 ± 0.04 for Disruptol-B) than in the DMSO (229.89 ± 4.29) (Fig. 3).

**Fig. 4:**
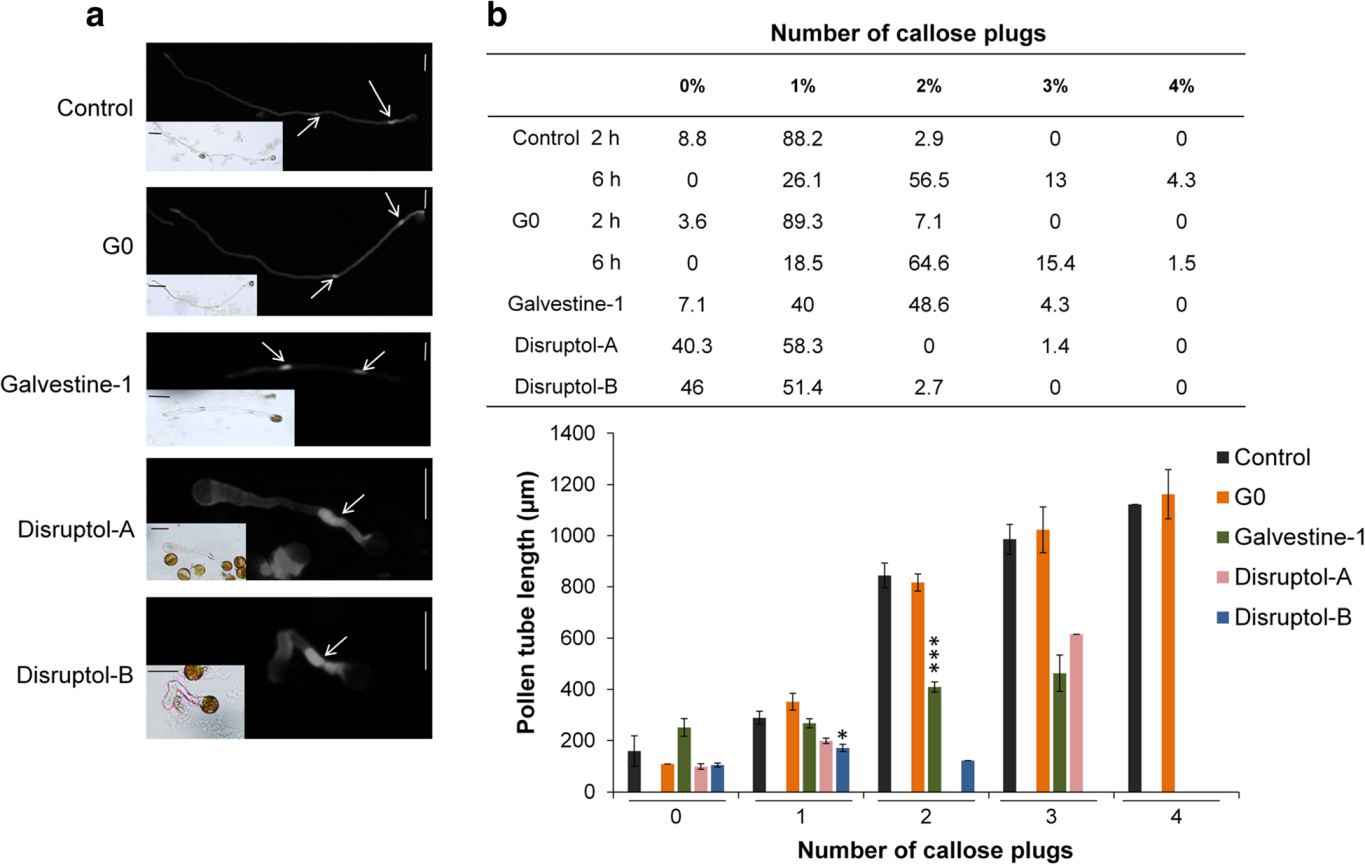
Effect of the compounds on callose plug deposition after 6 h of treatment. **a** Cytochemical staining of callose with DAB showing callose plugs (white arrows). **b** Distribution (%) of the number of callose plugs in treated pollen tubes and its relation with the mean length of pollen tubes. Data represent the mean (40 ≤ *n* ≤ 98 pollen tubes) from two biological replicates ± SEM. For the controls (DMSO and G0), data are a combination of pollen tubes grown for 6 h and 2 h, in order to get shorter pollen tubes. Asterisks indicate significant differences (*** *P* < 0.001, ** *P* < 0.01 and * *P* < 0.05) from the control DMSO 0.3% according to pairwise comparison using Wilcoxon’s test analysis with Holm adjustment with R software according to batch of callose plugs number dataset. Scale bar = 40 μm

### Effect of the compounds on cell wall deposition

To investigate the effect of the compounds on the number of callose plugs and callose deposition in the cell wall, we used decolorized aniline blue (DAB) (Fig. 4a). It is important to note that pollen tubes grown for 2 h in the control conditions (DMSO and G0) were added to the data set in order to be able to compare the number of callose plugs in short tubes (i.e. 6 h of growth for treated pollen tubes). Pollen tubes grown for 2 h with DMSO and G0 were less than 500 μm long and for most of them (~ 90%), they produced one callose plug (Fig. 4b). After 6 h, control pollen tubes showed normal callose deposition in the cell wall and plugs (most of them contained 2 plugs, the remaining pollen tubes had 1 or 3 callose plugs and only few < 5% had 4 callose plugs) (Fig. 4b). The first callose plug was close to the pollen grain (Fig. 4a), the other ones were regularly deposited and their numbers increased with the gain of pollen tube length (Fig. 4b).

When pollen tubes were treated with Disruptol-A, the length of pollen tubes ranged between 48 and 579 μm and contained 0–1 callose plug for 98.6% of them (Fig. 4b). Treatment with Disruptol-B induced a decrease of the pollen tube length (200 μm maximum) but the tubes still contained up to 1 callose plug (Fig. 4b). It is relevant to note that, in certain cases, galvestine-1, Disruptol-A and Disruptol-B treatments induced also callose deposition in the cell wall at the tips of the tubes for 0.7, 7.5 and 2.7%, respectively. This pattern of deposition was absent in the controls. Pollen tubes treated with galvestine-1 were shorter than in G0 but still contained a high number of callose plugs between 1 and 3 for tubes reaching 200 and 400 μm, respectively (Fig. 4b). For the same average length (~ 400 μm), pollen tubes treated with galvestine-1 contained 2 (for most of them) or 3 plugs.

**Fig. 5:**
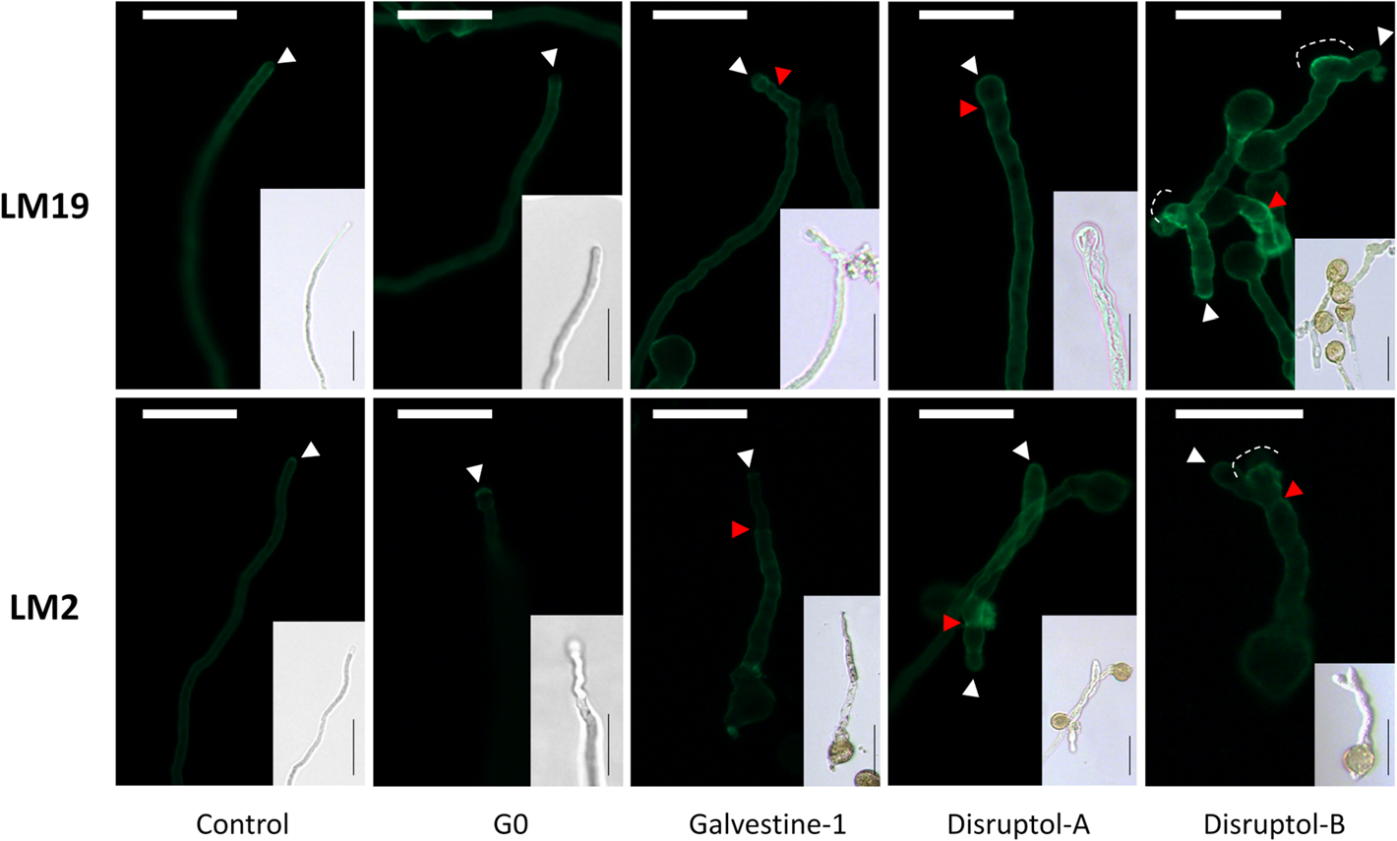
Cell surface immuno-labeling of epitopes associated with weakly methylesterified homogalacturonan and arabinogalactan proteins using LM19 and LM2, respectively on pollen tubes treated with the compounds for 6 h from 2 biological replicates. White arrow head = pollen tube tip, red arrowhead = ring-like deposit and dashed white line = pollen tube deformation. Scale bar = 40 μm

Immunolabelling of pollen tubes with the LM19 antibody (Fig. 5), which recognizes low methylesterified HGs, revealed that this epitope was mostly detected in the shank of pollen tubes in the control conditions (DMSO and G0). The treated pollen tubes showed different distributions of this epitope. 85% of pollen tubes treated with galvestine-1 showed a localization at the tip, compared to the control (14%). For 100% of those treated with Disruptol-A, the epitope recognized by LM19 was localized in the entire pollen tube cell wall and 92% for the pollen tubes treated with Disruptol-B. A brighter labelling was also observed in pollen tube distortion zones which originally were the expanding tip that stopped to grow leading to the emergence of a new tip. With all the treatments, brighter ring-like deposits were also observed and in the swollen tips of pollen tubes treated with Disruptol-A suggesting an accumulation of cell wall material during the slow growth phases.

Immunolabelling with the LM2 antibody, which recognizes epitopes associated with AGPs, revealed that this epitope was brightly labelled at the tip of control pollen tubes (DMSO and G0). 93.7% of pollen tubes treated with galvestine-1 and 100% of pollen tubes treated with Disruptol-A did not show a brighter labeling at the tip compared to the control (20%). All the treatments also induced brighter ring-like deposits along the pollen tube cell wall as observed with LM19.

### Effect of the compounds on actin dynamics and RIC4, a tip-polarized marker

**Fig. 6:**
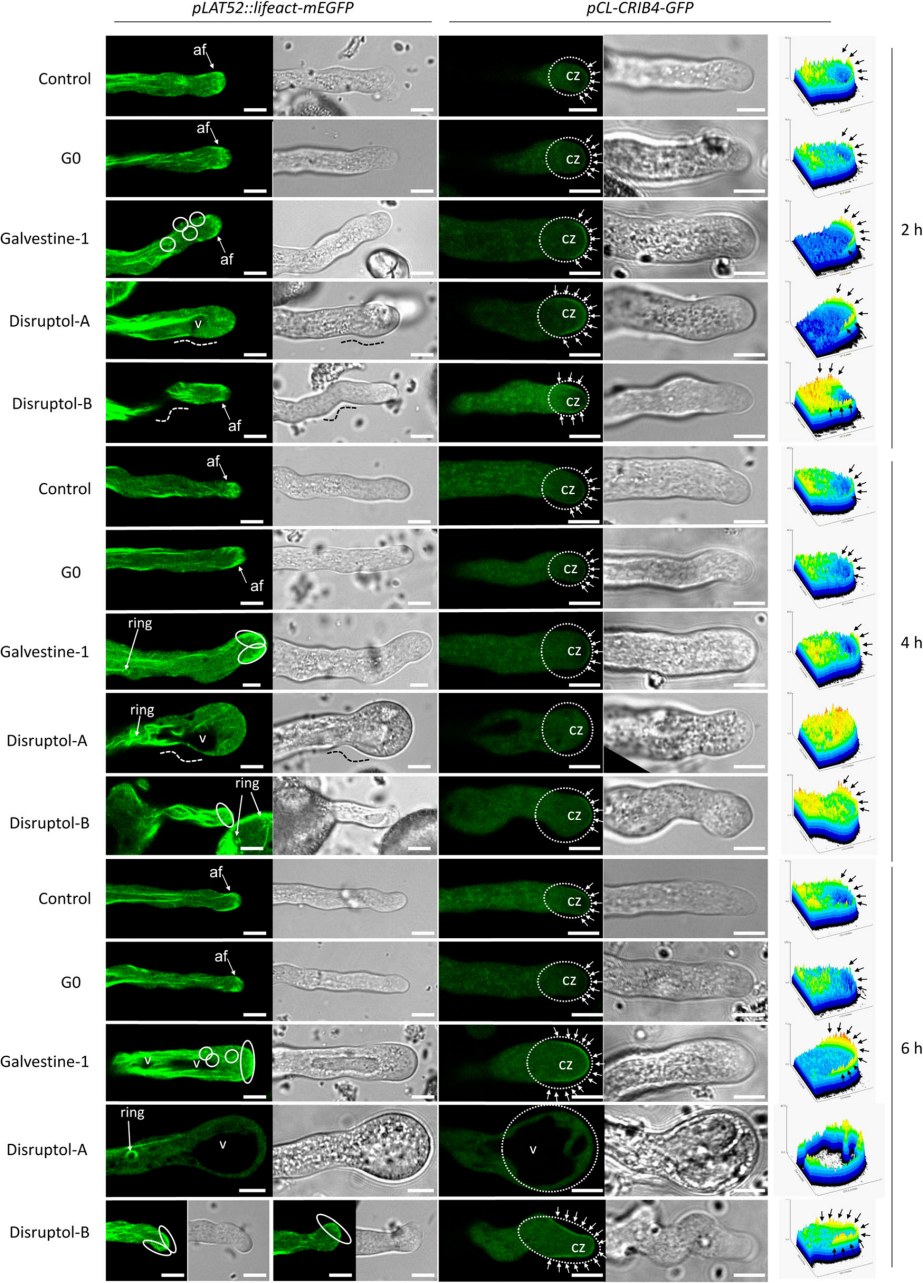
Effect of the compounds on actin filament and RIC4 dynamics using pollen tubes expressing *Lifeact-mEGFP* and *CRIB4-GFP* from 2 biological replicates after 2, 4 and 6 h of growth. On the right: 3D construction (pixel intensity) of RIC4 distribution in the pollen tube tip. af = actin fringe, CZ = clear zone, v = vacuole. Dashed white circle = pollen tube tip, dashed white line = pollen tube deformation, oval white circle = actin fringe accumulation, small arrow = RIC4 location at the plasma membrane and white circle = small punctate actin foci. Scale bar = 5 μm

To investigate whether the miss-localization of the cell wall polymers and the growth defect were due to a disruption of the intracellular trafficking, actin filament dynamics was followed using pollen tubes expressing *pLAT52::lifeact-mEGFP*. In the controls (DMSO and G0), after 2, 4 and 6 h of incubation, actin dynamics was normal with long cables observed in the shank region of the pollen tube and the actin fringe at the tip (Fig. 6; Additional file 2: Figure S2; Additional file 3: Video S1). These structures remained highly dynamics and pollen tubes were expanding normally overtime (Additional file 2: Figure S2). After 4 h and 6 h of treatment with Disruptol-A, vacuoles that are normally located in the back of the pollen tube were clearly visible in the tip region (Fig. 6, Additional file 2: Figure S2, Additional file 4: Video S2) and the actin fringe begun to disappear with the appearance of the swollen tip and was totally absent after 6 h of incubation (Fig. 6). At this time, pollen tubes were not expanding anymore but actin cables were still moving (Additional file 2: Figure S2). After 2, 4 and 6 h of incubation with Disruptol-B, the actin fringe and the spatial organization of actin filaments also looked abnormal (Fig. 6). However, after 4 and 6 h, pollen tubes were still expanding but more slowly than the control and in a winding way (Additional file 2: Figure S2; Additional file 5: Video S3) and no vacuoles were detectable at the tip (Fig. 6; Additional file 2: Figure S2). After 2 h and 4 h of incubation with galvestine-1, pollen tubes were also expanding but after 6 h, the growth was reduced (Additional file 2: Figure S2). After 4 h, the shape of the actin fringe changed with the increasing size of the pollen tube diameter. It was located in the subapical region and at the beginning of the shank region. After 6 h of treatment, the actin fringe was more detectable in the apex and disappeared from the shank region and the subapical region (Fig. 6). Small punctates of actin foci were also observed in the shank close to the apical region only in pollen tubes treated with galvestine-1. Disruptol-A and galvestine-1 treatments induced the appearance of actin rings after 4 and 6 h of incubation in the shank region. Actin rings were also observed in pollen grains treated with Disruptol-B. Actin rings were not observed in the pollen tubes of the control and G0 (Fig. 6).

The effect of the compounds on ROP1, an apical growth marker, was assessed by following the location of RIC4, an effector protein of ROP1 using *pCL::CRIB4-GFP*. In the controls (DMSO and G0), RIC4 localization remained normal after 2, 4 and 6 h of growth (Fig. 6) with pulsatile appearance/disappearance at the plasma membrane in the apical dome (Additional file 6: Figure S3). Under all conditions and in all pollen tubes, GFP was detected in the cytoplasm.

After 2 h of treatment with Disruptol-A, a perturbation of RIC4 dynamics was observed. RIC4 was not only localized at the apical dome but also in the subapical region and sometimes this location was persisting (Fig. 6; Additional file 6: Figure S3). After 4 h of incubation, RIC4 localization disappeared from the apical plasma membrane and GFP was only located in the cytoplasm (Fig. 6). RIC4 localization at the tip of pollen tubes treated with Disruptol-B was extremely versatile and depended on the pollen tube shape. RIC4 was localized in the subapical region and then moved from one side to the other side of the apical dome of the pollen tube. This was associated with a slow growth and multiple changes of the tip growth direction (Fig. 6 and Additional file 7: Figure S4).

After 2 and 4 h of treatment with galvestine-1, RIC4 was located, as in DMSO and G0 conditions, in the apical region (Fig. 6). The location was expanded to the subapical region after 6 h of treatment with galvestine-1 (Fig. 6).

## Discussion

### The compounds disrupt actin dynamics and ROP signaling: two central mechanisms in pollen tube tip-growth

The actin cytoskeleton plays a critical role in pollen tubes and polar growth [19] and the treatments with galvestine-1, Disruptol-A and Disruptol-B were able to disrupt actin cytoskeleton dynamics. Treatment with Disruptol-A induces a progressive disappearance of the actin fringe which completely disappears after 6 h and the appearance of the phenotype and pollen tube growth reduction. Similar effects were observed with the membrane trafficking inhibitor Brefeldin A (BFA) or with the electron transport chain inhibitor potassium cyanide (KCN) [22, 55]. The treatments with Disruptol-B and galvestine-1 did not induce the disappearance of the actin fringe but led to a disruption of the structure. The instability of the actin fringe with Disruptol-B treatment may explain the winding shape of the pollen tube. Indeed, it was shown that the turnover of actin filaments (polymerization/depolymerization) was an early event for pollen tube directional growth changes [56].

All the treatments induced ring-shaped actin structures in the shank of pollen tubes. These structures that represent a circular conformation of actin filaments were observed in most pollen tubes and were already described in several studies using either staining techniques or expressing fluorescent protein-tagged actin-binding domains [57–62]. Even if the high level of Lifeact expression can induce artefact due to the competition with native actin binding proteins [59, 63], it is possible that the ring-shaped actin structures in treated pollen tubes reflect a certain physiological state in the cell such as heat stress, calcium availability and extracellular pH changes [60, 64].

All treatments disturb RIC4 location and oscillation compared to the control. In pollen tubes treated with Disruptol-A, RIC4 completely disappeared from the apical region and this was accompanied by the disappearance of the actin fringe. These results are correlated with the fact that RIC4 pathway promotes the assembly of the actin fringe at the tip of the pollen tube [28].

Galvestine-1 is an inhibitor of the biosynthesis of galactolipids, more precisely MGDG. These galactolipids are critical for the biogenesis of photosynthetic membranes, and they act as a source of polyunsaturated fatty acids for the whole cell in phosphate shortage [54]. The expression of genes involved in galactolipid synthesis was shown to be strongly activated during pollen development and germination suggesting their importance during pollen maturation and pollen tube growth [65]. Galactolipids are present all along the membrane of pollen tubes [54] and inhibiting the biosynthesis of galactolipids may affect the plasma membrane ROP signaling/location. Interestingly, small and rare punctate actin foci were observed with galvestine-1 treatment. These structures are unusual and poorly described in the literature. Nevertheless, it appears that actin foci are induced during self-incompatibility (SI) response in *Papaver* [66] and are able to change the intracellular location of two actin-binding proteins, cyclase-associated protein and actin-depolymerizing factor [67]. Therefore, we postulate that the lipid homeostasis changes at the plasma membrane caused by galvestine-1, alter ROP1 and actin localization and dynamics [23–26, 68]. Thus, the disruption of actin filaments and dynamics by the treatments may result from an inhibition or an activation of actin binding proteins (ABPs) which play a crucial role in actin polymerization and depolymerization. In fact, the construction of actin structures requires several ABPs [58] such as formin, an actin-nucleating protein, implicated in establishing the subapical actin [69] or villin, a major bundling factor stabilizing actin filaments [55].

### The compounds disturb cell wall distribution and lead to a modification of pollen tube morphology

The number of callose plugs is generally correlated with the length of pollen tubes [36, 70]. Treatment with LatB (Latrunculin B), an inhibitor of actin polymerization, affects callose plug deposition in pollen tubes and induces callose deposits at the tip. Based on the location of the callose, it has been postulated that LatB may trigger a stimuli response that enhances callose synthesis by activating callose synthases located at the tip [71]. In our experiments, when pollen tubes are treated with galvestine-1, Disruptol-A or Disruptol-B, the pollen tube length is not correlated with the number of callose plugs. In fact, the pollen tube length decreases but the number of callose plugs remained unchanged, suggesting that callose plugs are not pollen tube length-dependent but are synthesized in a time-dependent manner. But this needs further investigation. Even if actin filaments are critical for the distribution of callose synthases [34] and that previous studies have demonstrated that a disorganized cellulose synthase complex in the plasma membrane may be capable of catalyzing the synthesis of both cellulose and callose [72, 73], it is difficult to link the effect of our molecules to callose plugs formation or deposition. The synthesis of callose at the tip may be a protective mechanism of the pollen tube to recover from the treatment as shown/suggested with the emergence of new tip on treated pollen.

The appearance of the phenotype, especially with Disruptol-A and Disruptol-B treatments, and the inhibition/reduction of the elongation suggest that the cell wall deposition is affected by the treatments. In untreated pollen tubes, weakly methylesterified HG epitopes are mostly present in the shank but not at the tip, in contrast arabinogalactan protein-associated epitopes are more detectable at the tip. These results are in agreement with previous studies [31, 32, 74, 75]. It is generally established that pectins are secreted highly methyesterified at the extreme apex, and are then de-esterified through the activity of pectin methylesterases within the cell wall [76]. This tight regulation is thought to control the stiffness of the cell wall between the tip (extensibility) and the shank (rigidity) to sustain the cylindrical shape of the tube [77, 78]. All the treatments show an abnormal tip localization of LM19 antibody binding. Pectin remodeling might thus be affected by the treatments impacting the capacity of the pollen tube cell wall extension [37, 71, 79]. The absence of tip-localized labelling of AGPs in the presence of Disruptol-A and galvestine-1 confirmed that cell wall material delivery was altered as AGPs are assumed to be involved in the deposition of new cell-wall material during pollen-tube growth [74, 75]. As actin cytoskeleton is important for vesicle trafficking, the modification of the cell wall polymer distribution is possibly related to an alteration of vesicular transport and polarized exocytosis of cell wall polymers and cell wall-modifying enzymes [80–82]. Blocking AGPs at the plasma membrane surface of pollen tubes by treatment with the Yariv phenylglucoside also induced callose deposition at the tip, mislocation of AGPs and pectins along with growth arrest [74, 83]. Interestingly, the effect was reversible upon removal of the reagent and a new emerging tip could be produced back from the original tip that was blocked [74] as it is observed with Disruptol-B.

Besides their role as second messenger in countless physiological processes often associated with cytotoxicity [84–87], ROS are also known to act on wall plasticity [88] and are implicated during pollen tube growth [12, 13]. During this study, ROS production was assessed using NBT, which reacts specifically with O_2_^•-^. The production of O_2_^•-^ significantly decreased only with Disruptol-A and Disruptol-B (Fig. 3). O_2_^•-^ plays a role in pollen tube growth and reorientation [12, 89]. ROS may also play a role in influencing actin polymerization dynamics in plant cells [90]. We can hypothesize that the disruption of the O_2_^•-^ production by Disruptol-A and Disruptol-B can then induce actin dynamics disruption. The compounds used in this study affect more clearly the actin-fringe. As with KCN, the fringe is among the first components to degrade together with the loss of the clear zone and the inhibition of growth, but with a continuation of cytoplasmic streaming [22]. ROS are known to also play roles in modulating cell wall extensibility to allow expansion but also to prevent tip bursting [91]. Following Disruptol-A and Disruptol-B treatments, the decrease of O_2_^•-^ level induces tip swelling and the loss of polarity, probably by altering the balance between different forms of ROS that promotes cell wall stiffening and/or loosening [92].

However, the links between the modification of polarized growth, the remodeling of cell wall polymers and actin dynamic remain unclear and will await further investigation.

## Conclusion

### Possible targets for Disruptol-A and Disruptol-B

**Fig. 7:**
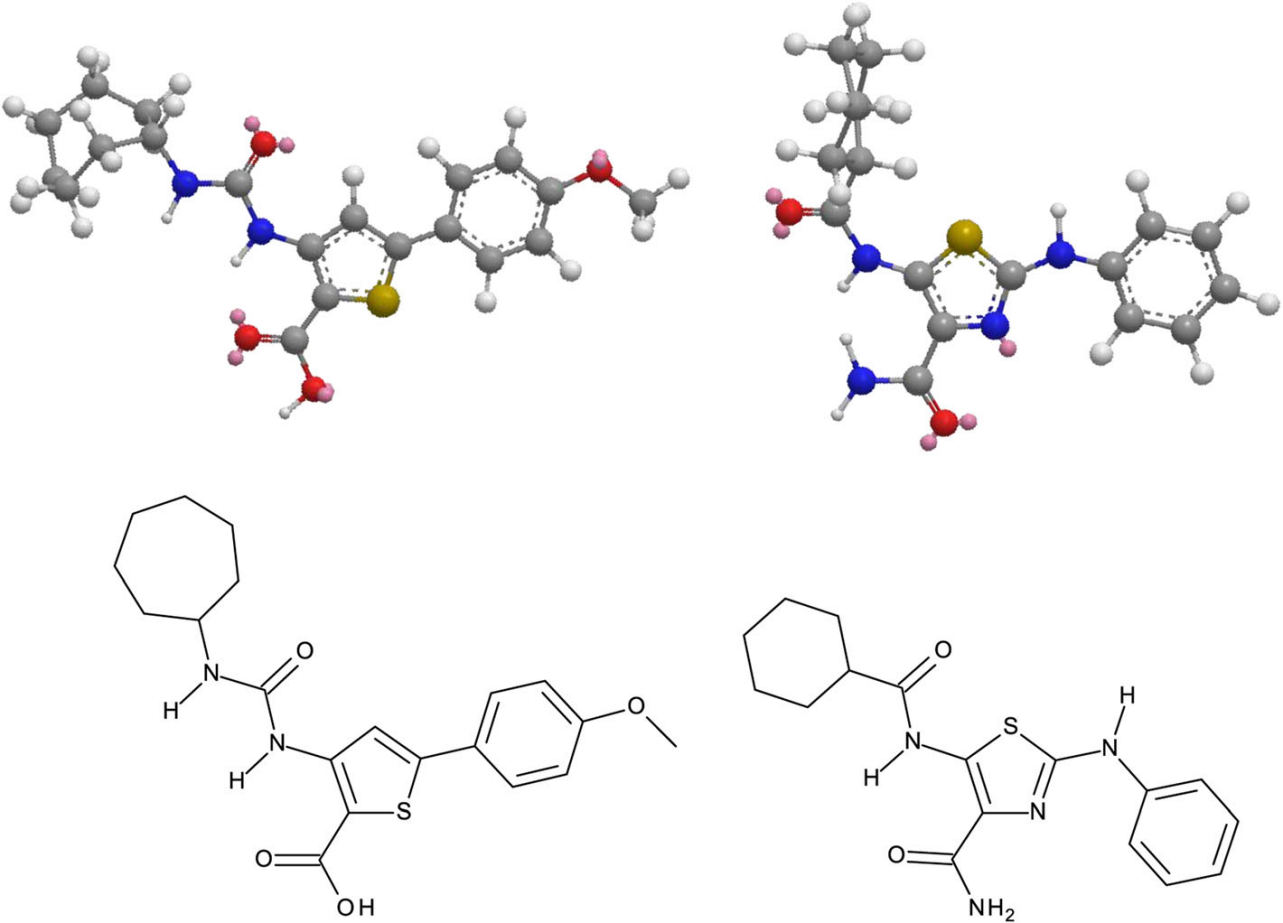
3D and 2D representations of Disruptol-B (Left) and UNC-3230 (right)

The targets of Disruptol-A and Disruptol-B are currently unkown. Disruptol-A and Disruptol-B are two synthetic compounds from the CERMN’s chemolibrary. From a structure-activity relationship (SAR) point of view, Disruptol-A and Disruptol-B theoretically exhibit appropriate structural features to engage inhibitory interactions with kinases as illustrated by the molecules, ureidofurane and thiophene carboxamides known to interfere with the inhibitor of nuclear factor kappa-B kinase (IKK) [93]. More specifically, the pharmacophoric features of Disruptol-B are closely related to those present in the structure of UNC3230, a PIP5K inhibitor (Fig. 7) [94] and this lipid kinases family could be potential targets for our compounds. Further investigations are required to verify this hypothesis and determine the direct and indirect effects of Disruptol-A and Disruptol-B on the tip-growth machinery.

Phosphoinositides together with actin dynamics are crucial for maintenance of vacuole morphology [95]. Recently, Arabidopsis *vac14* loss-of-function mutant, deficient in AtVAC14, a homolog of the yeast and metazoan VAC14 was implicated in the synthesis and turnover of Phosphatidylinositol 3–5 bi-phosphate (PI(3–5)P_2_) was characterized [96]. In Arabidopsis, VAC14 is constitutively and strongly expressed in developing pollen and its loss leads to pollen abortion and pollen tube growth defect [96]. Moreover, treatment of pollen tubes with the cell permeable inhibitor of PIPkinase, YM-201636, induced similar defects as observed with the treatments with Disruptol-A and B i.e. reduction of pollen germination and shorter tubes with wider diameter [96]. Similarly, overexpression in tobacco pollen tubes of PIP5K10 or PIP5K11, two pollen specific phosphatidylinositol-4-phosphate (PI4P) 5-kinases, able to produce in vitro phospholipid phosphatidylinositol-4,5-bisphosphate resulted in severe tip swelling, vacuolization and altered actin fine structure [97]. We might then hypothesized that Disruptol-A interferes with phosphoinositide metabolism in pollen tubes perturbing the actin cytoskeleton which can be influenced by PI4P 5-kinases and possibly contributing to the control of the pool of plasma membrane-associated Nt-Rac5. In fact, Nt-Rac5 is a member of the small GTPase family implicated in actin reorganization during cell growth [98]. Orthologs named ARAC4/ROP2, ARAC1/ROP3, ARAC6/ROP5, ARAC11/ROP1 and ARAC3/ROP6 are found in the Arabidopsis genome and ARAC11/ROP1 have been implicated in pollen tube growth [29]. The disruption of RIC4 location with Disruptol-B and galvestine-1 treatments is also accompanied with the disorganization of the actin fringe. RIC4 oscillates from the tip to the sub-apical tip region and its location predicts pollen tube directional growth suggesting that the abnormal localization of RIC4 in the apical region explains the multi-directional growth of pollen tubes [28]. Although, it remains unclear whether RIC4 directly or indirectly interacts with actin filaments by regulating actin polymerization factors [98], our results showed that disruption of RIC4 dynamics is closely related to the formation of the actin fringe and confirmed that apical filamentous actin dynamics and concentrations are influenced by ROP-GTPases [27].

## Methods

### Plant growth conditions

Seeds of *A. thaliana* Col-0 (stored at 4 °C) were spread on the surface of sterilized soil and cultivated in growth chambers in long-day photoperiod (16 h light/8 h dark at 20 °C/16 °C, respectively) and 60% relative humidity. Seeds containing the *pLAT52::lifeact-mEGFP* [17, 99] or *pCL-CRIB4-GFP* [100] constructions were a gift of Pr. Shanjin Huang (Institute of Botany, Chinese Academy of Sciences, Beijing, China) and Pr. Zhenbiao Yang (Center for Plant Cell Biology, University of California, Riverside, USA), respectively.

*Nicotiana tabacum* cv. Xanthi seeds were spread on the surface of sterilized soil. Plants were grown with a photoperiod of a 16 h light/8 h dark cycle at 25 and 22 °C during the light and dark period, respectively. Relative humidity was maintained at 60%, and plants were watered every 2 d. Tobacco seeds were a gift from UMR 6037 (CNRS, Université de Rouen, Rouen, France).

*Solanum lycopersicum var. cerasiforme* ‘West Virginia 106’ (wva106) seeds were sown 1 cm under the surface of sterilized soil and cultured in a growth chamber. Plants were grown with a photoperiod of a 16 h light/8 h dark cycle at 25 and 22 °C during the light and dark period, respectively. Relative humidity was maintained at 60%, and plants were watered every 2 d. Tomato seeds were a gift from Dr. Pierre Baldet (INRA, Université de Bordeaux, UMR 1332, Bordeaux, France).

### Pollen tube culture

Pollen grains of *A. thaliana* were germinated in liquid germination medium (GM) containing 5 mM CaCl_2_ 2H_2_O, 0.01% (*w*/*v*) H_3_BO_3_, 5 mM KCl, 1 mM MgSO_4_ 7 H_2_O, and 10% (w/v) sucrose, pH 7.5 as described previously [101]. As recommended by [102], 40 fully open flowers collected in the second row from the top of the primary and secondary inflorescences (in 1.5 mL Eppendorf® plastic tube) were submerged in 1 mL of GM. Tubes were shaken with a vortex to release the pollen grains from the anthers. Flowers were removed with a pair of tweezers and pollen suspension was then centrifuged at 4000 *g* for 7 min. New GM was added to the pellet and pollen were transferred in 96-well-plates (ThermoFisher®) or in μ-slides 18-well (Ibidi®) to be observed under an inverted microscopeor a confocal inverted microscope respectively and grown at 22 °C in the dark.

Pollen grains were collected from freshly dehisced anthers, and the stamens of three flowers (tobacco) or five flowers (tomato) were submerged in 5 mL of BK medium [1.62 mM H_3_BO_3_, 1.25 mM Ca (NO_3_)_2_, 4H_2_O, 2.97 mM KNO_3_ and 1.65 mM MgSO_4_, 7H_2_O] [103] containing 15% sucrose. Pollen grains were suspended in the GM by vortex, and the stamens were removed with tweezers. Tomato pollen tubes were grown in glass vials at 22 °C in the dark for 6 h under agitation. Tobacco pollen tubes were grown in 24-well-plates (ThermoFisher®), without agitation at 22 °C in the dark.

Pollen grains were considered as germinated when the length of the tube was longer than the diameter of the pollen grain. When burst, smaller pollen tubes were considered as “early burst” whereas longer pollen tubes were considered as “lately burst”. When no apparent tube tip was observed, pollen grains were considered as non-germinated.

### Chemical screen

A set of 258 compounds, representative of the chemical diversity (centroids approach) of the chemical library of the CERMN (Centre d’Etudes et de Recherche sur le Médicament de Normandie, Normandie Univ, UniCaen, France), was screened. Compounds were solubilized in 100% DMSO at 10 mM, aliquoted and stored at − 80 °C. For the primary screen, compounds were used at 20 μM (diluted in GM) and selected for their abilities to induce pollen tube morphology. For the primary screen, the negative control was 0.1% DMSO. Molecule screening and the dose-response effects were performed in 96-well-plates.

Two compounds were then selected that we named Disruptol-A and Disruptol-B (Fig. 1a). Another compound (galvestine-1) was also used in the study (Fig. 1a). Galvestine-1 is known to reduce pollen tube length in vitro [54]. The negative control of galvestine-1 was G0. G0 possess a galvestine-1 structure modification that leads to the loss of bioactivity (Fig. 1a) [54].

The dose-effects of the compounds were tested on pollen grains, as described above with concentrations ranging from 0.5, 1, 5, 10, 20 and 30 μM. Compounds at 10 mM in DMSO were diluted in distilled water to reach a concentration of 500 μM. Different volumes of DMSO 5%, the compound at 500 μM and GM were mixed to get a final DMSO concentration of 0.3% for all the treatments.

### Cytochemical staining

#### Callose staining

Decolorized aniline blue in 100 mM K_2_HPO_4_ 1% pH 12 was used to localize callose [102]. Decolorized aniline blue was directly added to the medium after 6 h of culture at a final concentration of 0.1%. The observation was performed after 2 h of incubation in the dark at room temperature.

#### Superoxide detection

Superoxide anion (O_2_^•-^) was detected using nitro blue tetrazolium (NBT; Alfa Aesar®). NBT solution was prepared in GM at 7 mM and incubated for 20 min with pollen tubes at a final concentration of 1.2 mM [92].

### Immunolocalization of Arabidopsis pollen tube cell wall epitopes

Two primary monoclonal antibodies (LM19 and LM2, PlantProbes) were used. The mAb LM19 recognizes epitopes associated with low methylesterified homogalacturonan (HG) domain of pectin [104] whereas LM2 was generated against rice AGPs and recognizes a carbohydrate epitope containing a β-linked glucuronic acid [105].

After 6 h of culture, a fixation medium containing PIPES 100 mM, 4 mM MgSO_4_ 7H_2_O, 4 mM EGTA, 10% (*w*/*v*) sucrose and 5% (*v*/v) of paraformaldehyde pH 7.5 was added to the GM and incubated overnight at 4 °C. Pollen tubes were centrifuged at 4000 *g* for 7 min. The pellet was suspended in 200 μL of Phosphate Buffer Saline (PBS: 137 mM NaCl, 2.7 mM KCl, 7 mM Na_2_HPO_4_ 2H_2_O, 1.5 mM KH_2_PO_4_ pH 7.2) and then deposited on a μ-Slide VI ^0.4^ Poly-L-lysine (Ibidi®), incubated overnight at room temperature to fix the pollen tubes on the lysine matrix and then rinsed 3 times with PBS before being incubated with PBS 3% fat-free milk for 30 min at room temperature. Pollen tubes were then rinsed 3 times with PBS. Primary antibodies were diluted at 1:5 with PBS and incubated overnight at 4 °C in the dark. Pollen tubes were rinsed 3 times with PBS and the secondary anti-rat antibody combined with fluorescein isothiocyanate (FITC; Sigma) diluted at 1:50 with PBS was incubated for 3 h at 30 °C. Negative controls were carried out by omitting the primary antibody. Before observation, pollen tubes were rinsed three times with PBS and suspended in a mix of citifluor/PBS (v/v). Observations were made as indicated below.

### Microscopy and image acquisition

A confocal inverted microscope Leica SP2 was used to observe pollen tubes expressing *pLAT52::lifeact-mEGFP* and *pCL-CRIB4-GFP* using a μ-slide 18-wells (Ibidi®). Images were acquired every 2 s 915 msec for 5 min. Different filter sets were used, *pLAT52::lifeact-mEGFP* (absorption 488 nm, emission 505–545 nm); *pCL-CRIB4-GFP* (absorption 488 nm, emission 498–560 nm).

An inverted microscope Leica DMI 6000B was used to observe phenotypes under bright field or epi-fluorescence for aniline blue and FITC with different filter sets (absorption, 405 nm; emission, 523 nm or absorption, 460–500 nm; emission, respectively).

The program ImageJ [106] was used to determine manually on each picture the pollen tube germination rate, abnormal pollen tube shape, pollen tube length and diameter. For ROS quantification with NBT, pictures were transformed as grayscale images, a mark was drawn from the tip to 30 μm behind and the surface was manually selected using freehand selection tool. The mean value of the pixel intensity was obtained with the measure tool.

### Statistical analyses

Data were treated with R software [107] and represent the mean ± SEM. Pairwise comparison were performed with the Dunnett’s test or with Wilcoxon’s test analysis with Holm adjustment according to parametric and non-parametric batch of dataset. Significant differences were ^*^ *P* < 0.05, ^**^ *P* < 0.01 and ^***^ *P* < 0.001.

## Additional files

Additional file 1: **Figure S1.** Dose-response effect of the compounds on (a) *Solanum lycopersicum* and (b) *Nicotiana tabacum* pollen tubes after 6 h of culture. (PDF 3876 kb)

Additional file 2: **Figure S2.** Time-lapse observations of actin dynamics in *Arabidopsis thaliana* pollen tubes treated for 2, 4 and 6 h with the compounds. (PDF 220 kb)

Additional file 3: **Video S1.** Time-lapse imaging of actin dynamics in untreated *Arabidopsis thaliana* pollen tubes after 6 h of growth. A frame was acquired every 3 s during 5 min. (MP4 538 kb)

Additional file 4: **Video S2.** Time-lapse imaging of actin dynamics in *Arabidopsis thaliana* pollen tubes treated for 6 h with Disruptol-A. A frame was acquired every 3 s during 5 min. (MP4 967 kb)

Additional file 5: **Video S3.** Time-lapse imaging of actin dynamics in Arabidopsis thaliana pollen tubes treated for 6 h with Disruptol-B. A frame was acquired every 3 s during 5 min. (MP4 668 kb)

Additional file 6: **Figure S3.** Time-lapse imaging of RIC4 dynamics in Arabidopsis thaliana pollen tubes treated for 2, 4 and 6 h with the compounds. (PDF 717 kb)

Additional file 7: **Figure S4.** Time-lapse imaging of RIC4 dynamics of a pollen tube treated with Disruptol-B for 2 h. (PDF 169 kb)

## Abbreviations

AGPs: arabinogalactan proteins
CDC42: Cell division control protein 42
CRIB: Cdc42/Rac-interactive binding
DMSO: Diméthylsulfoxyde
HG: Homogalacturonan
IKK: Inhibitor of nuclear factor kappa-B kinase
LM: Leeds Monoclonal
MGDG: Monogalactosyldiacylglycerol
NADPH oxidase: β-nicotinamide adenine dinucleotide phosphate oxidase
NBT: Nitroblue tetrazolium
PBS: Phosphate buffer saline
PIP5K: Phosphatidylinositol-4-phosphate (PI4P) 5-kinases
RBOH: Respiratory burst oxidase homolog
RIC4: ROP-interactive CRIB motif-containing protein 4
ROP1: Rho-related GTPases in plants 1
ROS: Reactive oxygen species
