# Supplementary material for: A chemical screen identifies two novel small compounds that alter Arabidopsis thaliana pollen tube growth
Source: BMC Plant Biol. 2019 Apr 22;19:152. doi: 10.1186/s12870-019-1743-9 (PMC6475968; doi:10.1186/s12870-019-1743-9)
Supplement: Supplementary file 1 — Figure S1. Dose-response effect of the compounds on (a) Solanum lycopersicum and (b) Nicotiana tabacum pollen tubes after 6 h of culture. (PDF 3876 kb) [file 12870_2019_1743_MOESM1_ESM.pdf]

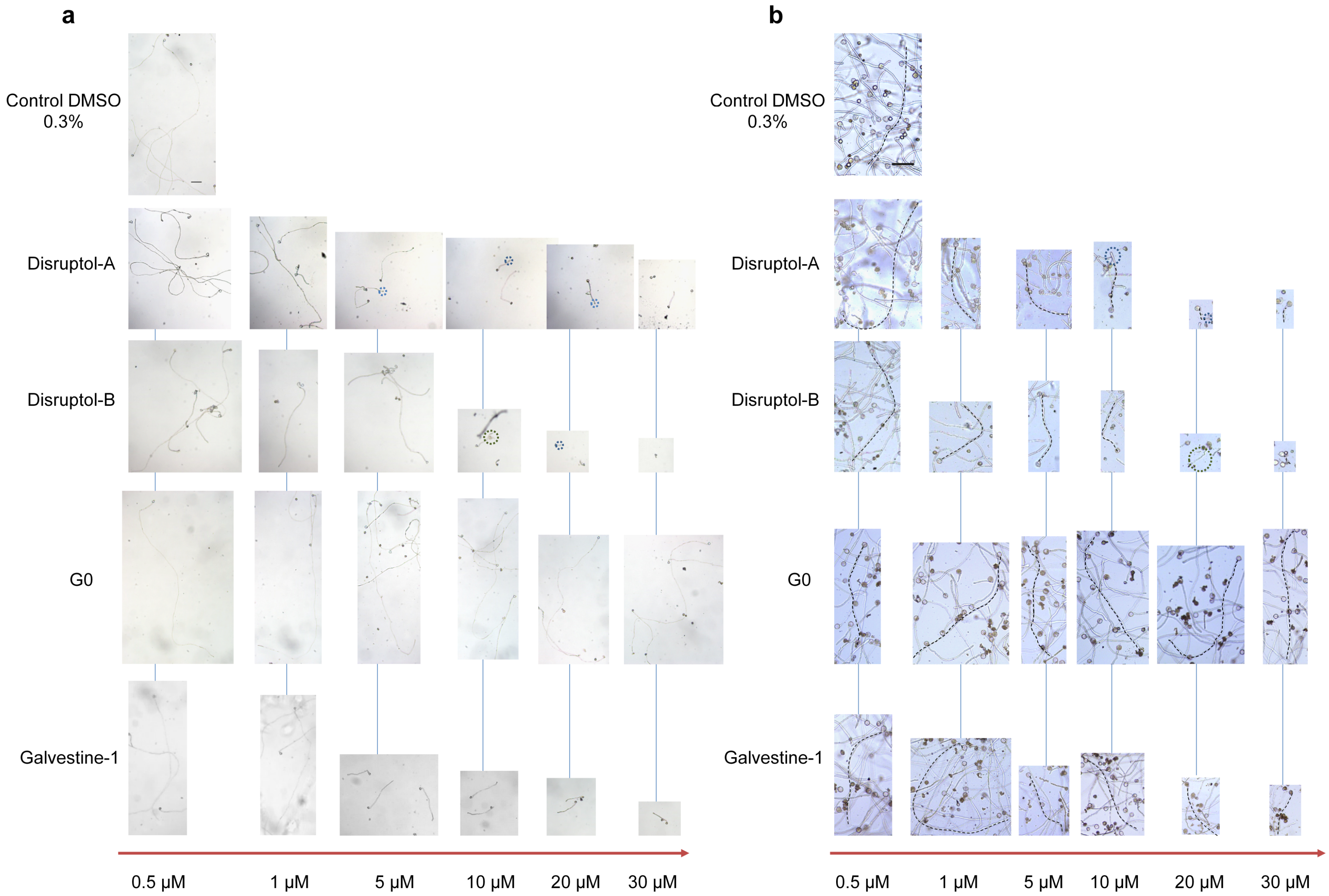
a
b
Control DMSO 0.3%
Disruptol-A
Disruptol-B
G0
Galvestine-1
0.5 μM
1 μM
5 μM
10 μM
20 μM
30 μM

Additional File 1: Figure S1. Dose-response effect of the compounds on (a) *Solanum lycopersicum* and (b) *Nicotiana tabacum* pollen tubes after 6 h of culture. Dashed black line = pollen tube. Dashed blue circle = swollen tip. Dashed green circle = pollen tube deformation. Scale bar = 100 µm.
