# Supplementary material for: A chemical screen identifies two novel small compounds that alter Arabidopsis thaliana pollen tube growth
Source: BMC Plant Biol. 2019 Apr 22;19:152. doi: 10.1186/s12870-019-1743-9 (PMC6475968; doi:10.1186/s12870-019-1743-9)
Supplement: Supplementary file 2 — Figure S2. Time-lapse observations of actin dynamics in Arabidopsis thaliana pollen tubes treated for 2, 4 and 6 h with the compounds. (PDF 220 kb) [file 12870_2019_1743_MOESM2_ESM.pdf]

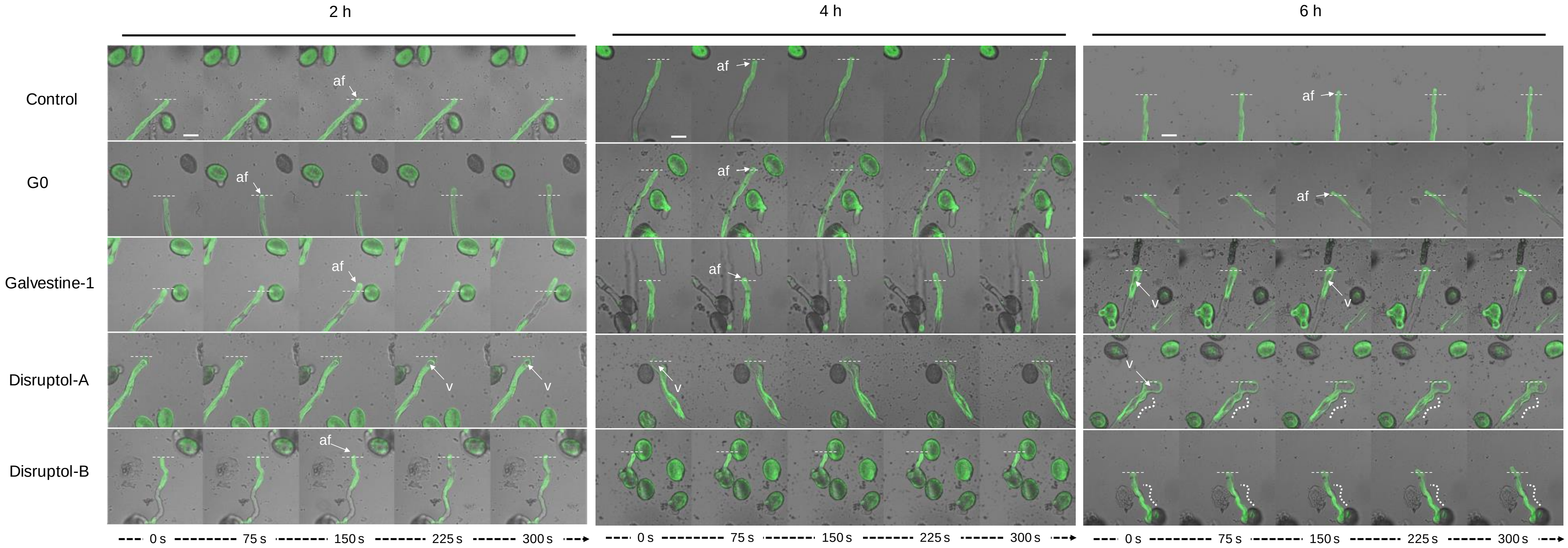

Additional file 2: Figure S2. Time-lapse imaging of actin dynamics in *Arabidopsis thaliana* pollen tubes treated for 2, 4 and 6 h with the compounds. af = actin fringe, v = vacuole. Dotted lines indicate pollen tube tip position at 0 sec. Scale bar = 10 µm.
