# Supplementary material for: A chemical screen identifies two novel small compounds that alter Arabidopsis thaliana pollen tube growth
Source: BMC Plant Biol. 2019 Apr 22;19:152. doi: 10.1186/s12870-019-1743-9 (PMC6475968; doi:10.1186/s12870-019-1743-9)
Supplement: Supplementary file 6 — Figure S3. Time-lapse imaging of RIC4 dynamics in Arabidopsis thaliana pollen tubes treated for 2, 4 and 6 h with the compounds. (PDF 717 kb) [file 12870_2019_1743_MOESM6_ESM.pdf]

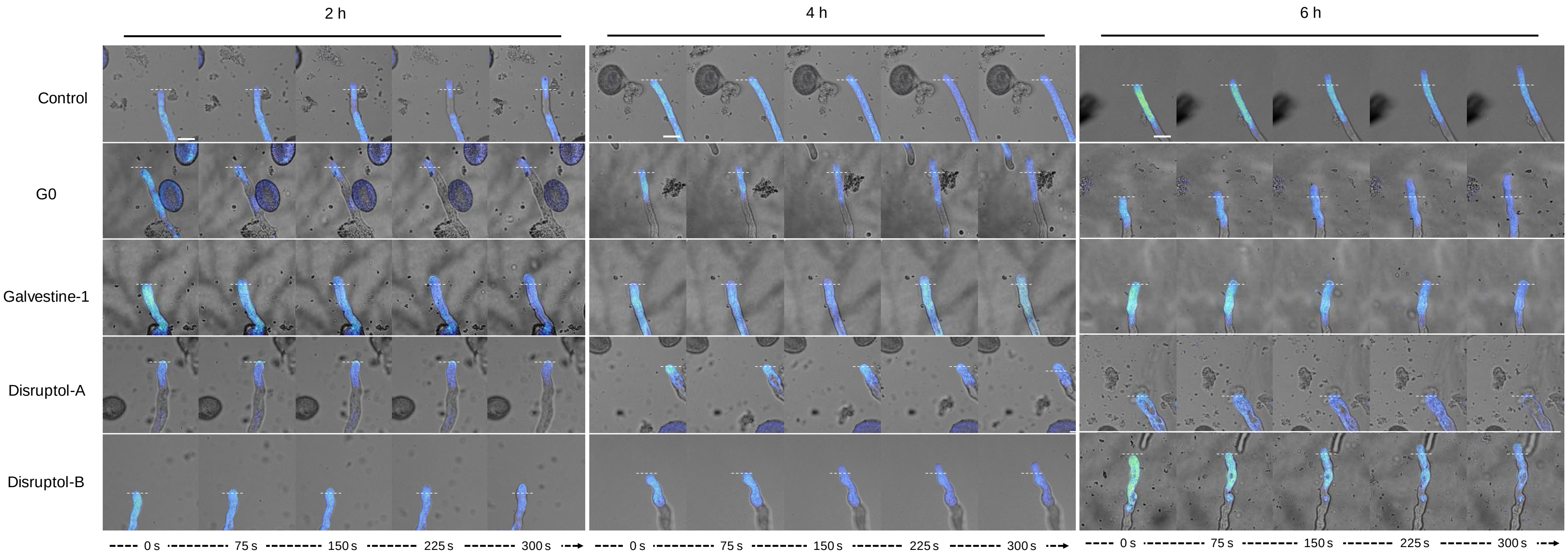

Additional file 6: Figure S3. Time-lapse imaging of RIC4 dynamics in *Arabidopsis thaliana* pollen tubes treated for 2, 4 and 6 h with the compounds. Dotted lines indicate pollen tube tip position at 0 sec. Scale bar = 10 µm.
