# Supplementary material for: A chemical screen identifies two novel small compounds that alter Arabidopsis thaliana pollen tube growth
Source: BMC Plant Biol. 2019 Apr 22;19:152. doi: 10.1186/s12870-019-1743-9 (PMC6475968; doi:10.1186/s12870-019-1743-9)
Supplement: Supplementary file 7 — Figure S4. Time-lapse imaging of RIC4 dynamics of a pollen tube treated with Disruptol-B for 2 h. (PDF 169 kb) [file 12870_2019_1743_MOESM7_ESM.pdf]

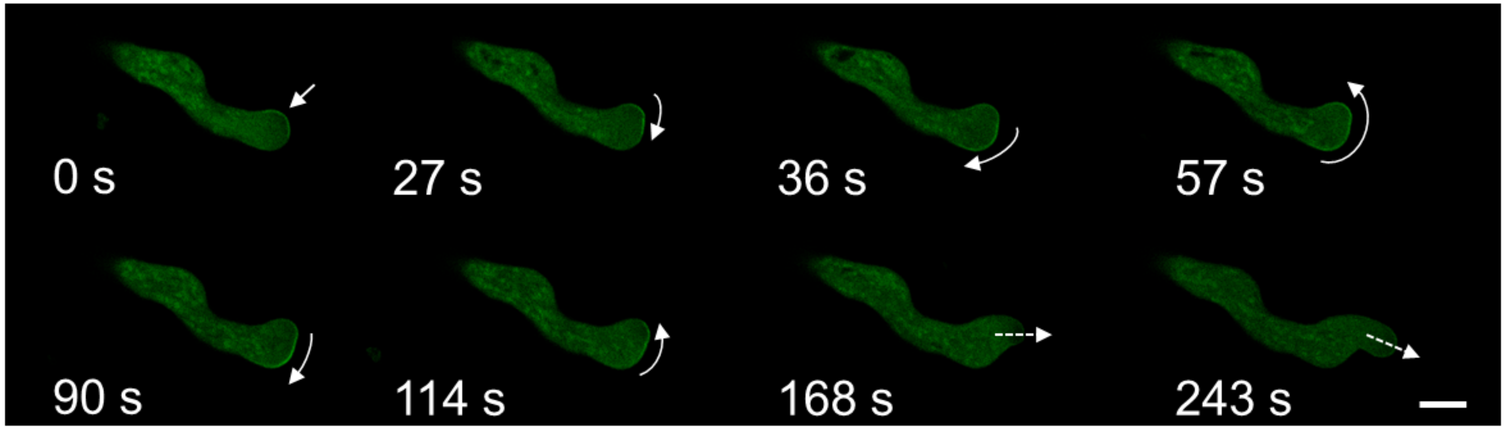

Additional File 7: Figure S4. Time-lapse imaging of RIC4 dynamics of a pollen tube treated with Disruptol-B for 2 h. Arrow indicates RIC4 localisation and movement. Dashed arrow indicates pollen tube growth direction. Scale bar = 5 µm.
